# The cerebellum and cognitive neural networks

**DOI:** 10.3389/fnhum.2023.1197459

**Published:** 2023-07-28

**Authors:** Pingshu Zhang, Liqin Duan, Ya Ou, Qirong Ling, Lingyun Cao, Hongchun Qian, Jian Zhang, Jing Wang, Xiaodong Yuan

**Affiliations:** ^1^Department of Neurology, Kailuan General Hospital, North China University of Technology, Tangshan, Hebei, China; ^2^Key Laboratory of Neurobiological Function in Hebei Province, Tangshan, Hebei, China

**Keywords:** cerebellum, cognitive functions, brain networks, visuospatial, attention, execution, working memory, language

## Abstract

Cognitive function represents a complex neurophysiological capacity of the human brain, encompassing a higher level of neural processing and integration. It is widely acknowledged that the cerebrum plays a commanding role in the regulation of cognitive functions. However, the specific role of the cerebellum in cognitive processes has become a subject of considerable scholarly intrigue. In 1998, Schmahmann first proposed the concept of “cognitive affective syndrome (CCAS),” linking cerebellar damage to cognitive and emotional impairments. Since then, a substantial body of literature has emerged, exploring the role of the cerebellum in cognitive neurological function. The cerebellum’s adjacency to the cerebral cortex, brainstem, and spinal cord suggests that the cerebral-cerebellar network loops play a crucial role in the cerebellum’s participation in cognitive neurological functions. In this review, we comprehensively examine the recent literature on the involvement of the cerebellum in cognitive functions from three perspectives: the cytological basis of the cerebellum and its anatomical functions, the cerebellum and cognitive functions, and Crossed cerebellar diaschisis. Our aim is to shed light on the role and mechanisms of the cerebellum in cognitive neurobrain networks.

## 1. Introduction

Cognitive function represents a complex neurophysiological capacity of the human brain, encompassing a higher level of neural processing and integration. According to a systematic analysis conducted by Lo et al. (2019), which included a total of 3,146 stroke patients across 8 countries from 13 different studies, it was found that 44% of the patients experienced cognitive impairment. The cerebrum is involved in various cognitive functions, including attention, executive function, memory, language, and visuospatial abilities. For example, research has found that ischemic lesions in regions such as the frontal lobe, temporal lobe, and parietal lobe are closely associated with post-stroke cognitive impairments. Related studies have also mapped out lesion-symptom profiles. For instance, visual-spatial function is associated with the right temporal-parietal cortices and subcortical networks in both hemispheres. Attention function is linked to the ventral attention system and white matter tracts in both hemispheres. Executive and memory functions are associated with networks comprised of the left hemisphere cortex and subcortical regions. Language function is related to cortical regions in the left hemisphere, including the left inferior frontal gyrus, left middle temporal gyrus, left middle occipital gyrus, and left angular gyrus (Zhao et al., 2018; Weaver et al., 2021). In recent studies, it has been reported that an imbalance at the neural network level is among the factors contributing to cognitive dysfunction (Birle et al., 2021). The frontal, temporal, and parietal cortices encompass several brain networks that are linked to cognition, including the frontoparietal network, the orbitofrontal anterior temporal network, the frontotemporal network, and the posterior parietal cortical network (Jung et al., 2018). We are intrigued by the interplay between the cerebellum and cognitive function, considering its role as a subcortical structure intricately connected by extensive neural fibers to the cerebral cortex, brainstem, and spinal cord.

From an evolutionary standpoint, studies have indicated a relatively consistent ratio between the number of neurons in the cerebral cortex and the cerebellum, with an average of 3.6 across the 19 species analyzed. When significant evolutionary changes occur in the number of neurons in the cerebral cortex, there is a corresponding adjustment in the number of neurons in the cerebellum, suggesting a self-organizing mechanism. The cortico-ponto-cerebellar projection emerges as a plausible mechanism for facilitating the numerical congruence between neurons in the cerebral and cerebellar cortices (Herculano-Houzel, 2010). Furthermore, it is worth noting that the development of the cerebellum precedes that of the cerebral cortex, as Purkinje cells in the cerebellum begin to form prior to 8 weeks post conception (pcw). In contrast, the peak of neurogenesis in the cerebral cortex takes place around 16 pcw (Keefe and Nowakowski, 2020). In a recent prospective follow-up study conducted by Erdal et al. (2021), a cohort of 23 patients diagnosed with isolated cerebellar infarction and 22 healthy volunteers were recruited. The primary objective of the study was to assess cognitive function in both the acute phase and at least 6 months post-stroke. The research findings indicated that cerebellar lesions had a significant negative impact on cognitive function (Erdal et al., 2021). Kalashnikova et al. (2005) demonstrated that a considerable proportion of patients (88%) diagnosed with cerebellar infarction exhibited cognitive neurological deficits. These deficits encompassed attentional decline in 83% of cases, executive decline in 80% of cases, visuospatial deficits in 72% of cases, memory decline in 68% of cases, and language deficits in 65% of cases (Kalashnikova et al., 2005).

Cerebellar damage frequently gives rise to a diverse range of cognitive dysfunctions. In light of this, our hypothesis posits that the cerebellum exerts influence on specific cerebral regions via the cerebro-cerebellar loop, thereby contributing to cognitive dysfunction. Several research methods, including neuropsychology, electroencephalography (EEG), and functional magnetic resonance imaging (fMRI), have effectively delineated the specific brain regions accountable for cognitive functions, as well as the interconnections between these regions, thereby establishing the brain network. In their study, Wang et al. (2019) employed fMRI to identify abnormal alterations in the bilateral middle cingulate, paracingulate, posterior cingulate, and precuneus lobes. These findings suggest that these specific brain regions may serve as pivotal contributors to cognitive impairment following cerebellar infarction. Moreover, prior investigations have established a correlation between dysfunction of the cerebro-cerebellar networks and compromised interactions among critical brain regions, such as the right frontal cortex, left hippocampus, and right cingulate gyrus (Fan et al., 2019). The cerebro-cerebellar loop primarily consists of the cortex-pontine-cerebellum bundle and the cerebellum-thalamus-cortex bundle. This loop serves as an efficient pathway for transmitting information from the cerebral cortex to the cerebellum. Subsequently, the cerebellum processes this information and provides feedback to the cerebral cortex. Therefore, gaining a comprehensive understanding of the anatomical correlations between the cerebellum and cortex facilitates a deeper comprehension of the functional associations between the cerebellum, the brain, and its involvement in various cognitive neurological functions (Baumann et al., 2015).

Contemporary research has revealed the involvement of the cerebellum in a multitude of functions, encompassing motor, cognitive, emotional, and social domains (Koziol et al., 2014; Adamaszek et al., 2017; Li et al., 2021). The cerebellum governs motor functions, including the regulation of body balance, muscle tone, and the coordination of voluntary movements. In addition to its role in motor functions, the cerebellum also plays a significant role in emotional learning, pain processing, and the expression of verbal emotions, among other functions (Adamaszek et al., 2017). Besides, the cerebellum plays a crucial role in goal-directed social navigation, emphasizing its priority in coordinating and facilitating social interactions and navigation toward specific social goals (Li et al., 2021). Studies as early as 1992 have demonstrated that cerebellar lesions can result in cognitive function alterations, highlighting the role of the cerebellum in cognitive processes (Ivry and Baldo, 1992). However, it was not until that Schmahmann and Sherman (1998) conducted a comprehensive series of studies specifically focusing on 20 cases of patients exclusively diagnosed with cerebellar injury. Their investigations revealed significant impairments in various cognitive functions, including visuospatial abilities, attention, executive function, memory, and speech, among patients with cerebellar injury. They have collectively summarized these cognitive deficits as “cognitive affective syndrome.” These studies propose that disturbances in the regulation of neural circuits connecting the prefrontal, posterior parietal, superior temporal, and limbic cortices with the cerebellum may underlie cerebellar cognitive dysfunction. These studies represent a significant milestone in the investigation of cerebellar cognitive function (Schmahmann and Sherman, 1998). Nevertheless, it is important to consider certain limitations in Schmahmann’s study, including the restricted sample size, the passage of time, the comparatively less advanced neuropsychological and neuroimaging techniques available at that time, the limited understanding of how the cerebellum is intricately involved in the regulation of cognitive functions, and the lack of detailed description regarding brain-cerebellar networks.

Recent research has presented compelling evidence regarding the impact of cerebellar lesions on the cerebrum. However, a comprehensive exploration is still needed to unveil the precise influence of the cerebellum on the cerebrum and the specific cognitive functions that are affected. Previous investigations have primarily focused on examining the cognitive functions associated with individual brain regions while neglecting the crucial interplay among these regions within neural networks. Thus, the objective of the present study is to address this gap by investigating the neural networks that connect the cerebellum and the cerebrum.

## 2. The cytological basis of the cerebellum and its anatomical functions

From a cytological perspective, the cerebellum is comprised of two main types of cells: cerebellar cortical neurons and deep cerebellar neurons. Deep neurons are primarily involved in basic muscle coordination, whereas neurons in the cerebellar cortex are responsible for regulating, connecting, and maintaining higher cognitive functions, such as memory. Mossy fibers serve as excitatory afferent fibers within the cerebellum. They transmit information from the spinal cord, pontine nucleus, and reticular nucleus of the brainstem to the granule cells in the granular layer. The granular layer also contains Golgi cells, which secrete GABA transmitters to inhibit the activity of granule cells. Climbing fibers, another type of excitatory afferent fiber, form direct synapses with the dendrites of Purkinje cells. They play a critical role in learning and working memory in humans. The molecular layer of the cerebellum houses two types of inhibitory interneurons: astrocytes and basket cells. These interneurons have axons that form synapses with Purkinje cell dendrites, thereby regulating the activity of Purkinje cells. Purkinje cell axon terminals predominantly terminate within the cerebellar nuclei, representing the sole efferent fibers originating from the cerebellar cortex. The cerebellar function relies significantly on the orchestrated activity of granule cells and Purkinje cells (Zhu et al., 2020). Granule cells serve as the exclusive excitatory neurons responsible for relaying input information from mossy fibers to Purkinje cells (Rhee et al., 2021). Purkinje cells play a pivotal role in regulating cerebellar function as key elements in the cerebellar circuitry (Nóbrega et al., 2013). Bailey et al. (1998) conducted a study examining the histopathological findings in the brains of six patients with autism and intellectual disability. Their observations revealed a decrease in the number of cerebellar Purkinje cells in all six patients, accompanied by gliosis. Chang et al. (2021) conducted a study involving Ankfy1 knockout mice and made significant observations. Their findings demonstrated that the knockout mice experienced a substantial loss of parvalbumin-positive cells within the Purkinje cell layer. These results strongly suggest that Ankfy1 knockout mice develop Purkinje cell degeneration, ultimately leading to motor dysfunction (Chang et al., 2021). Dickson et al. (2017) found that mice with >95% Purkinje cell loss had impaired reversal learning and extradimensional set-shifting, as reduced Purkinje cell numbers result in multiple changes in mPFC neurotransmitter dynamics, suggesting a mechanism through which cerebellar Purkinje cell loss may affect cognitive flexibility. Information from other neurons in the cerebellum converges on Purkinje cells and eventually projects via axons to the deep cerebellar nucleus, the only output neuron of the cerebellar cortex, whose loss leads to cerebellar ataxia (Dusart and Flamant, 2012; Cougnoux et al., 2020). Consequently, Purkinje cells, acting as the exclusive output neurons of the cerebellum, exert their influence not only on cerebellar motor function but also on cognitive function.

From an anatomical perspective, the cerebellum consists of the left and right hemispheres, along with the cerebellar vermis situated in the middle. It can be further divided into three lobes based on distinctive grooves and fissures: the anterior lobe, the posterior lobe, and the flocculonodular lobe. Alternatively, Schmahmann’s classification categorizes the cerebellum into 10 lobes, with lobes I through V comprising the anterior cerebellar lobe, lobes VI through IX forming the posterior cerebellar lobe, and lobes X constituting the flocculonodular lobe. Additionally, a particular study has revealed that lobule VII, which encompasses approximately 48% of the human cerebellar cortex and is located in the posterior lobe, exhibits an increase in size in correlation with higher cognitive abilities (Stoodley and Schmahmann, 2018; Lehman et al., 2020). By employing an ultra-high magnetic field, Serenos utilized human cerebellar specimens to perform scanning and reconstruct the cerebellar cortex. The calculated results of this study revealed that the surface area of the cerebellar cortex accounts for approximately 80% of the surface area observed in the cerebral cortex (Sereno et al., 2020). Despite its relatively small volume, the cortex of the cerebellum exhibits an extensive surface area, particularly in the posterior lobes. This significant cortical surface area prompts us to explore the relationship between the cerebellum’s function and that of the cerebrum.

Cerebellar lesions can occur in various locations, leading to distinct clinical manifestations in patients with cerebellar injury (Shin et al., 2017). Several different studies revealed that the anterior cerebellum was associated with the regulation of motor functions and the posterior cerebellum was involved in cognitive-emotional regulation. Therefore, the cerebellum could be divided into motor-cognitive dichotomies based on cerebellar anatomy (Stoodley et al., 2016; Shin et al., 2017; Schmahmann, 2019). Stoodley et al. (2016) used a voxel-based lesion-symptom mapping approach to assess motor and cognitive function in 18 patients with isolated cerebellar stroke and found that cerebellar lesions essentially did not affect cerebellar motor function as long as they did not involve the anterior cerebellum, which suggested that anterior cerebellar lesions caused cerebellar motor syndrome and posterior cerebellar lesions caused the cerebellar cognitive-emotional syndrome. In order to record changes in the resting-state network in the brain, Castellazzi recruited 22 healthy volunteers and exposed them to stories that activated particular cognitive functions. They were then scanned for resting-state functional fMRI at four time points before, during, and after the stories to record the changes in the network. This research showed that the posterior lateral cerebellum was connected to cognitive processing (Castellazzi et al., 2018). Three years later, Gok-Dursun described a patient who initially presented with psychomotor retardation in 2021 and was eventually diagnosed with “CCAS” using the CCAS scale test. His Magnetic Resonance Imaging (MRI) of the Brain revealed that he had bilateral posterior cerebellar infarcts with a predominance of the seventh and eighth lobes (Gok-Dursun et al., 2021). According to the studies mentioned above, the anterior and posterior cerebellar lobes perform different tasks.

In addition to having distinct anterior and posterior lobe functions, the cerebellum also exhibits some lateralization in the cognitive processes of its left and right hemispheres. The Addenbrooke’s Cognitive Test ACE III and the Pathway Test TMT were used to assess cognitive function in 31 patients with localized cerebellar lesions and 31 healthy volunteers. It was discovered that patients with left-sided cerebellar lesions were impaired in the attention and visuospatial domains, while patients with right-sided cerebellar damage had deficits in general cognitive function (Starowicz-Filip et al., 2021). The research by Baillieux also confirmed that the left cerebellum controlled attention and visuospatial skills, but it was also thought that the right cerebellar hemisphere influenced logical thinking and language processing skills (Baillieux et al., 2010). Yuan et al. (2022) used functional fMRI and transcranial direct current stimulation (tDCS) to reveal the different functions of the bilateral cerebellar lobules in bilingual language production, and they found that the right cerebellum was primarily involved in language control, the left cerebellum is mainly responsible for computational functions in language. Starowicz-Filip et al.’s (2021) study also concluded that the right cerebellum primarily innervates language function. Additionally, they discovered the existence of multiple connections between the dentate nucleus of the right cerebellar hemisphere and the language areas of the cerebral cortex, emphasizing the intricate relationship between these regions. Using diffusion tensor imaging, Kim et al. (2019) found dominant laterality of cerebellar afferent and efferent pathways in a right-handed population, with age-related correlation. Therefore, we speculate that the regulation of the cognitive function by dominant hand and age, which deserves further study.

The anatomical structure plays a crucial role in determining its function to a significant extent. The left and right hemispheres of the brain exhibit distinct functions, while demonstrating interconnections with the cerebellum, thereby forming cerebrum-cerebellum networks. Consequently, it can be ascertained that the left and right hemispheres of the cerebellum exhibit discrepant functions. Additionally, the cerebellar structure comprises anterior and posterior lobes, which are further subdivided into 10 lobules. Presently, research has solely elucidated the functions of select lobules within this complex. Nevertheless, with the extensive application and advancement of neuropsychology, neurophysiology, and fMRI, we anticipate the revelation of additional insights into the functionality of the cerebellar lobules.

## 3. Cerebellar and cognitive function

A large number of studies have shown that the cerebellum is involved in cognitive functions such as visuospatial, attention, execution, working memory, and language. Below we will discuss in detail which neural functions the cerebellum is involved in and the neural regulation mechanism of the Cerebro-cerebellar networks.

### 3.1. Cerebellum and visuospatial function

The capacity to assess and incorporate spatial data is referred to as the visuospatial function. As a result, defective spatial analysis or integration results in impaired spatial perception and behavior, which is what is meant by visuospatial dysfunction. Visuospatial dysfunction clinically usually includes impairments in visuospatial perception, visuospatial construction, and visuospatial memory (Salimi et al., 2018).

Cortical and subcortical regions are responsible for maintaining the function of visuospatial processing. Visual information normally travels from the retina through the thalamus to the occipital cortex (early visual cortex), from there it travels by ventral and dorsal fibers to the frontal cortex (Molinari et al., 2004; de Graaf et al., 2010). Molinari et al. (2004) discovered that cerebellar lesions can cause VSD in different dimensions and that the left and right cerebellar hemispheres are lateralized. To examine the impact of the cerebellum on the physical and digital line bisection task, Oliver et al. (2011) employed repetitive transcranial magnetic stimulation (rTMS) to temporarily inhibit cerebellar function in eight healthy participants. The selection of stimulation sites was based on the connectivity between the cerebellum and the contralateral prefrontal and parietal neural networks responsible for visuospatial function. The findings revealed that rTMS-induced inhibition of the cerebellum significantly impaired the participants’ performance in a digital spatial leveling linear task. This suggests that the cerebellum plays a crucial role in the processing of visuospatial information related to left and right spatial orientation (Oliver et al., 2011). In addition, a study that recruited 77 healthy volunteers to scan resting high-resolution NMRs to investigate the connectivity of the dentate nucleus with functional cerebral regions found that specific regions in the cerebellar dentate nucleus were connected to areas involved in visuospatial management in the frontoparietal occipital lobe (Guell et al., 2020). Sako’s study recently discovered that Parkinson’s disease (PD) patients’ cerebellar lobules were connected to the visuospatial network (Sako et al., 2021). When PD patients presented with visuospatial deficits, it was discovered that lobules VIII and IX of the cerebellar earthworm had improved functional connectivity with the default mode network (DMN), which could be explained by the cerebellum integrating visuospatial information through DMN to coordinate gait (Yin et al., 2021). The central brain regions comprising the default network primarily encompass the precuneus and the cingulate gyrus (Zhang et al., 2016). It is noteworthy that the precuneus is considered a component of the parietal lobe. Hence, when individuals with PD experience visuospatial dysfunction, it is plausible to speculate that the cerebellum may relay visual information to the frontal cortex by means of a pathway involving the thalamus, cingulate gyrus, and parietal lobe.

Recent research has unequivocally established the role of the cerebellum in visuospatial function, particularly in relation to the frontoparietal lobe. However, there is still insufficient evidence to pinpoint the exact cerebellar lobule that is involved. Consequently, exploring this aspect in greater detail could be pursued as a promising avenue for future investigations within this field.

### 3.2. Cerebellum and attentional function

According to cognitive neuroscience, the higher cognitive function of attention plays a crucial role in effectively allocating the brain’s processing resources to relevant stimuli (Fiebelkorn and Kastner, 2020). Posner and Petersen (1990) proposed the theory of attention networks. Neuroimaging studies investigating the spatial attention network have revealed that the frontal and parietal lobes serve as the sources of attention regulation prior to feedback reaching the visual cortex (Fiebelkorn and Kastner, 2020). The visual attention network consists of two subnetworks: the dorsal attention network (DAN) and the ventral attention network (VAN). Corbetta proposed that the DAN, comprised of the dorsal posterior parietal and frontal cortices, is involved in the cognitive selection of sensations and reactions. On the other hand, the VAN, composed of the temporoparietal and ventral frontal cortices, is responsible for recruiting sensory events and processing behavioral inputs (Corbetta and Shulman, 2002). Suo discovered that the functional network nodes of the DAN and VAN exhibit partial overlap with the anatomical network nodes. Specifically, they identified the posterior right inferior frontal gyrus, the anterior right middle frontal gyrus, and the superior right marginal gyrus as regions involved in these networks (Suo and Yu, 2019).

The cerebellum has been identified as a critical component in the functioning of visual attention, as demonstrated by studies conducted by Striemer et al. (2015) and Esterman et al. (2017). In the study conducted by Xie (2007), it was observed that individuals with cerebellar injury exhibited diminished orientation efficiency and impaired executive control when compared to the control group. These findings suggest that lesions in the cerebellum can specifically impair the orienting function of the attentional network. Craig et al. (2021) conducted a study examining attentional function in 14 patients with cerebellar infarction. The rationale behind this investigation was the potential functional association between damaged cerebellar regions and the dorsal and ventral attentional networks. The study revealed that patients with cerebellar damage exhibited impairments in the allocation of spatio-temporal attention (Craig et al., 2021). In a similar vein, Wang et al. (2021) enrolled 36 patients with posterior cerebellar infarction and 30 healthy volunteers for cognitive neurofunctional assessment and MRI scans to extract parameters related to the structural topological features of brain networks. The findings of this study indicated reduced global and local efficiency of brain networks, along with increased clustering coefficients and shortest path lengths within the patient group. The authors attributed the observed attentional dysfunction in the cerebellar infarction group to diminished connectivity efficiency within the cerebellar and default networks, as well as the frontotemporoparietal network (Wang et al., 2021). Furthermore, Mannarelli investigated changes in the amplitude of the P3b wave, which is indicative of alterations in attentional function, at various time points in a patient with left posterior lobe cerebellar infarction. The study revealed a decrease in early P3b wave amplitude, which the author attributed to the early dysfunction of cerebellar projections from the left posterior lobe to the prefrontal and posterior parietal cortices (Mannarelli et al., 2015).

In their study, Zhang et al. (2022) utilized resting-state fMRI studies to explore the relationships between spontaneous brain activity and response time to attentional tasks in children. They discovered significant correlations between brain activity in the bilateral middle frontal gyrus, default network, left precentral gyrus, left posterior cerebellar lobe, left thalamus, and right lingual gyrus. These findings suggest that the cortico-cerebellar-thalamo-cortical loop might serve as a potential neural basis for attentional executive function in school-age children (Zhang et al., 2022). Mannarelli et al. (2016) conducted a study involving the application of transcranial direct current stimulation (tDCS) to the cerebellum of healthy volunteers. During the experiment, novel stimulation paradigms were presented to the volunteers, while changes in brain potentials were recorded. This approach was employed due to the hyperpolarizing and neural excitability-reducing effects of cathodal tDCS, which can inhibit the output capacity of cerebellar Purkinje cells. Consequently, the inhibition of cerebellar function may lead to dysfunction within the ventral attentional brain network. The results of the study demonstrated that cathodal cerebellar tDCS resulted in a decrease in P300 amplitude (Mannarelli et al., 2016). Additionally, in another study by Mannarelli, a cohort of 29 healthy volunteers was recruited to undergo either anodal, cathodal, or sham tDCS. Attentional networks were assessed before and after tDCS stimulation, revealing that cathodal cerebellar tDCS led to a decrease in the efficiency of the executive attentional network. One possible explanation for this finding is that the cerebellum indirectly processes errors through the regulatory function of a cortical area involved in perceiving conflict signals. Moreover, the perception of errors requires the involvement of multiple cortically relevant regions, including the cingulate cortex, lateral prefrontal cortex, and inferior parietal cortex (Mannarelli et al., 2019).

The aforementioned studies provide evidence supporting the involvement of the cerebellum in the attentional network. Nevertheless, the precise connectivity between the cerebellum and the dorsal and ventral attentional networks remains unclear. It is hypothesized that the cerebro-pontocerebellar-cerebellar loop may play a crucial role in this regard, and its exploration can be facilitated through the use of diffusion tensor imaging. By employing such methods, a more comprehensive understanding of the anatomical connections between these networks can be achieved.

### 3.3. Cerebellum and executive functions

Executive Functions (EF) is a conceptual framework that encompasses a diverse array of sub-cognitive neural functions, including but not limited to working memory, inhibitory control, cognitive flexibility, planning, reasoning, and decision-making (Cristofori et al., 2019).

The involvement of the cerebellum in the regulation of EF remains a topic of ongoing debate and lacks consensus among researchers. Beuriat et al. (2020) conducted a study in which three different groups were selected to undergo EF tests. Interestingly, the group with cerebellar injury showed similar performance to the healthy control group across five specific executive function subtests. These findings raise questions about the widely held belief regarding the critical involvement of the cerebellum in the regulation of EF. Beuriat attributed the disparity between their study’s results and previous studies to the composition of their subject pool. Unlike previous studies that primarily included individuals in the acute phase of cerebellar disease, the authors intentionally focused on subjects in the late phase of cerebellar damage. This choice shed light on the potential plasticity and compensatory properties of the cerebellum, suggesting that the cerebellum may exhibit adaptive changes in response to damage (Beuriat et al., 2020). According to the findings of Shin et al. (2017), patients with lesions specifically in the right posterior-intermediate lobar region of the cerebellum demonstrated significant deficits in executive function. Notably, their performance on executive function tests, such as the Korean Lexicality Test, Digit Symbol Coding, and the Korean Connectedness Test A, was notably impaired. These results strongly indicate that the right posterior-intermediate cerebellar lobe plays a crucial role in the execution of executive function tasks (Shin et al., 2017). In the study conducted by Liu et al. (2021), it was discovered that the preservation of executive function in patients with mild cognitive impairment is contingent upon the integrity of the executive network. Additionally, the researchers observed increased fractional amplitude of low-frequency fluctuation in the cerebellar vermis among patients with impaired executive function. These findings strongly suggest the involvement of the cerebellar vermis in the modulation of executive function (Liu et al., 2021). In a pioneering study, Allen et al. (2005) provided the initial evidence of functional coherence between the human cerebellar dentate nucleus and the parietal and prefrontal cortices, utilizing fMRI techniques. These findings strongly indicate the presence of functional connections between the cerebellum and the parietal as well as prefrontal cortices, highlighting the potential role of cerebellar-parietal and cerebellar-prefrontal networks in human brain function (Allen et al., 2005). Using fMRI, D’Esposito et al. (1995) investigated the activation patterns of the prefrontal cortex in six healthy volunteers during dual-task work and single-tasking. Their findings revealed that the prefrontal lobe exhibited activation exclusively during dual-task operations, indicating its involvement as one of the central nodes within the central executive network. This study highlights the crucial role of the prefrontal cortex in the coordination and management of cognitive processes during multitasking scenarios (D’Esposito et al., 1995).

Multiple studies have consistently identified the frontal lobe as a crucial node within the executive control network, alongside the cerebellum. Nevertheless, the specific circuits and connections through which the cerebellum participates in the activation of the executive control network remain unclear. Considering this, there is a growing hypothesis that the pontine brain and thalamus may serve as crucial intermediaries in facilitating communication between the cerebellum and other components of the executive control network. Further research is warranted to explore these potential pathways and shed light on the intricate interactions between the cerebellum, pontine brain, thalamus, and the executive control network.

### 3.4. Cerebellum and working memory

Working memory plays a crucial role in various cognitive neuropsychological activities, including reading, comprehension, and learning. The term “working memory” was coined by Miller in 1960, and since then, both Miller and Baddeley have made significant contributions to its development. In 1974, Baddeley proposed the concept of working memory and has continued to refine it over the years. In his 2005 research, Baddeley likened working memory to a storage system, wherein information is initially perceived by the sensory system, temporarily stored in the limited-capacity short-term memory, and eventually transferred to long-term memory for permanent storage. Baddeley (2010) introduced a theoretical model of working memory, which consists of four components: the central executive system, the visuospatial sketchpad, the phonological loop, and the episodic buffer. Meanwhile, Miller suggested in 2018 that working memory serves two functions: information retention and the regulation of volitional processes (Miller et al., 2018).

The frontoparietal lobe, which serves as the primary cerebral cortex for the maintenance of working memory and as the source of top-down signals, has been extensively studied in relation to the regulatory mechanisms of working memory neural networks. It exerts influence on how working memory is processed in other cerebral cortex and subcortical regions (D’Esposito and Postle, 2015). Zhao et al. (2020) demonstrated that the frontal and parietal lobes are involved in the quantity control of working memory, while the lateral occipital complex (LOC) plays a role in precision control. Furthermore, an increased functional connection between the bilateral LOC and the right inferior frontal cortex was observed as the number of visual working memory items increased. This suggests that the link between accuracy-related and quantity-related cortical regions may be mediated by this connection (Zhao et al., 2020).

When a participant is exposed to an external stimulus, fMRI is commonly utilized to identify neural activity and the activation of functional regions in the relevant areas of the cerebral cortex. In a study conducted by Ashida et al. (2019), fMRI was used to scan 19 healthy volunteers while they performed a Stenberg task, a verbal working memory task, to investigate the brain areas that were active. Significant activation was observed in extensive regions of the cerebellar cortex during the encoding, maintenance, and retrieval stages of the working memory task. Further analysis using parametric models revealed a linear relationship between increasing verbal working memory load and the activity in bilateral cerebellar Crus I, right Crus II, right lobule VI, and VIII, with the highest activity observed in the right cerebellar hemisphere. These findings suggest that multiple areas of the cerebellum are involved in working memory processes (Ashida et al., 2019). Recent research conducted by Brissenden et al. (2021) has also provided new insights into the role of the cerebellum in the control of working memory (Stein, 2021). Brisenden conducted a study involving 16 healthy adult volunteers to investigate the role of working memory activation using fMRI. During a delayed motor direction recall task, blood oxygen level-dependent (BOLD) signals were measured in various brain regions. The results demonstrated that both the frontoparietal cortex and cerebellar lobule VIIb/VIIIa exhibited significantly higher BOLD signals during working memory tasks. Specifically, the subregion of lobule VIIb/VIIIa that showed preferential connectivity with the frontoparietal cortex exhibited content-specific activation during the delay period. These findings suggest that the frontoparietal cortex and cerebellar lobule VIIb/VIIIa play distinct roles in working memory activation and show correlated activity during fluctuations in information content (Brissenden et al., 2021). In a previous study by Ziemus, the involvement of the cerebellum in working memory was examined using fMRI. Unlike Brisenden’s study, Ziemus employed an n-back task and included nine patients with isolated cerebellar infarction along with nine healthy controls. During the performance of the n-back task, the activation patterns in different brain regions were analyzed in both groups. The findings revealed extensive activation in the cortico-cerebellar network, including the ventral lateral prefrontal cortex, dorsolateral prefrontal cortex, parietal cortex, anterior supplementary motor area, anterior cingulate gyrus, and medial cerebellum, in both the patient and control groups. Furthermore, the precuneus, angular gyrus, and posterior parietal lobes exhibited stronger activation, possibly compensating for the maintenance of cerebellar task performance in patients with cerebellar infarction (Ziemus et al., 2007).

To explore the relationship between the cerebellum and working memory, Seese conducted a comprehensive literature search using specific search phrases in MEDLINE and PubMed. The search focused on articles published between October 1, 2019, and January 8, 2020, encompassing systematic reviews, meta-analyses, thematic reviews, original research reports, and case reports. The study primarily targeted children as the study population, with a specific emphasis on acquired cerebellar diseases, cerebellar malformations, and genetic/developmental disorders of the cerebellum. This meticulous search aimed to facilitate a pooled analysis of existing research on the cerebellum and its involvement in working memory. Notably, working memory impairment emerged as a prevalent symptom in childhood cerebellar diseases, indicating the crucial role of the cerebellum in working memory function (Seese, 2020). In a previous case report by Nakamoto, a patient with acute cerebellar infarction exhibited clinical manifestations of episodic memory impairment. Single-photon emission computed tomography (SPECT) revealed insufficient perfusion in the right cerebellar hemisphere and the left frontotemporal area, including the left prefrontal lateral cortex, with slightly reduced perfusion in the left anterior thalamus. The authors suggested that the memory impairment was linked to the thalamus, which acts as a relay station in the complex network of cerebellar-brain connections (Nakamoto et al., 2015).

Based on the findings of these studies, it is proposed that the frontoparietal lobe, LOC, and cerebellum serve as primary brain regions involved in the regulation of the working memory network. Across different age groups, the cerebellum consistently demonstrates significant involvement in modulating working memory functions. Additionally, the close association between the cerebellar lobule VIIb/VIIIa and the frontoparietal brain network underscores their interconnected roles in working memory processes.

### 3.5. Cerebellum and language function

Language processing involves the collaborative contribution of both cortical and subcortical regions. Traditionally, language function was believed to primarily rely on the frontal cortex (Broca’s area) and the superior temporal cortex (Wernicke’s area) within the cerebral cortex (Friederici and Gierhan, 2013). However, recent attention has been directed toward the role of the cerebellum in language processing. The cerebellum’s involvement in language can be summarized as “timing, adaptation, and prediction,” as described by Moberget (Moberget and Ivry, 2016). While severe language impairment is less commonly observed in individuals with acquired cerebellar damage, subtle language dysfunction is frequently encountered in clinical observations. Furthermore, research has indicated that cerebellar abnormalities can affect various language-related functions such as planning, memory, fluency, and grammar (Marien et al., 2014).

Tani and Sakai proposed the idea that cerebellar lesions could disrupt speech, based on a case study of a male patient with neurogenic stuttering caused by cerebellar infarction. Interestingly, the patient did not exhibit stammering after thalamic hemorrhage and frontal infarction but developed stuttering following cerebellar infarction (Tani and Sakai, 2010). The neural network mechanisms underlying the cerebellar modulation of language are currently undergoing active exploration and investigation. Geva et al. (2021) conducted a study involving four individuals with right posterior cerebellar infarcts to examine their verbal fluency and sentence processing abilities using fMRI and neuropsychological techniques. The results revealed that three patients without infarction in the IX lobule recovered normal sentence processing after 1 year, whereas the patient with IX lobule infarction showed impaired sentence processing. The authors suggested that the IX lobule is involved in linguistic sentence processing and may be a component of the default mode network (DMN) (Geva et al., 2021). However, no research has yet explored the impact of DMN damage in cerebellar infarction patients on their speech abilities. Further investigation is needed in this area. In a study by Murdoch and Whelan (2007), language function was compared between 10 patients with left cerebellar stroke and 10 healthy volunteers. The results demonstrated that the cerebellar stroke group exhibited significantly lower scores on language variables compared to the control group. These findings suggest that left cerebellar hemisphere lesions may interfere with language processing, possibly due to frontal lobe underperfusion resulting from the cerebellar stroke (Murdoch and Whelan, 2007).

Reading is a fundamental aspect of language, and assessing reading ability typically involves employing various methods such as orthographic, phonological, and semantic tasks. Li et al. (2022) performed a study that demonstrated significant activation of the left lobule VII during the orthographic task, while the right Crus I and Crus II exhibited significant activation during the semantic task, as determined through voxel analysis. Additionally, region-of-interest analysis revealed significant activation in the medial or right portions of the right lobule Crus I during the phonological task. Through functional connectivity analysis, the study further revealed that the activated areas in the cerebellum associated with orthographic and semantic tasks exhibited functional connections with corresponding areas in the cerebrum. Similarly, the cerebellar areas involved in phonological processing were found to be functionally connected to the orthographic activation areas in the cerebrum. These findings collectively suggest a functional collaboration between the cerebrum and cerebellum during the reading process (Li et al., 2022). In order to investigate the activation patterns of language functions in the cerebrum and cerebellum, Lidzba conducted an fMRI study. The study involved recording the brain region activation in patients with congenital left hemisphere lesions and in healthy volunteers while performing the “word chain” task. The results revealed that the control group exhibited activation primarily in the left cerebral hemisphere and the right posterior cerebellar lobe. In contrast, the patient group exhibited greater activation in the right cerebral hemisphere and the left posterior cerebellar lobe. These findings suggest a reorganization of the cerebellar network involved in language production in individuals with congenital left hemisphere lesions (Lidzba et al., 2008). By conducting a comprehensive review of neuroanatomical, neuroimaging, and clinical studies, Marien and Borgatti (2018) propose that the cerebellum participates in an extensive array of non-motor language functions. They suggest that this involvement arises from a dense network of crossed and reciprocal connections between the cerebellum and various regions of the brain (Marien and Borgatti, 2018). Zhang provided a comprehensive summary of the potential mechanisms by which the cerebellum modulates language function, highlighting three key aspects. Firstly, the cerebellum may play a crucial role in integrating cortical information derived from language-related areas. Secondly, it is proposed that the cerebellum directly influences the cerebral cortex by transmitting signals to modulate language processing. Lastly, the cerebellum may indirectly enhance language function processing efficiency by modulating other cognitive functions. This analysis by Zhang offers valuable insights into the multifaceted role of the cerebellum in language modulation, shedding light on its involvement in various aspects of language processing (Zhang and Qiu, 2022).

The current body of research highlights the intricate involvement of the cerebellum in both motor and language functions. However, it remains uncertain whether the language function of the cerebellum represents an independent cognitive function or is closely intertwined with motor processes. It is important to note that motor abilities form the foundation of language, and extensive evidence from studies on individuals with cerebellar damage demonstrates impairments in both language and motor function. These findings from human research contribute valuable insights to our understanding of the role of the cerebellum in language function. Furthermore, it has been observed that bilateral damage to the cerebellum can result in language dysfunction. The disruption of the cerebral-cerebellar language network is considered to be the primary underlying cause of cerebellar language impairments. This highlights the critical role of the interaction between the cerebellum and cerebral regions in supporting language processing and suggests that disturbances in this network can significantly impact language function.

## 4. Crossed cerebellar diaschisis

Crossed cerebellar diaschisis (CCD) is characterized by a decrease in blood flow and metabolism in the contralateral cerebellum due to a unilateral supratentorial cerebral lesion. This phenomenon, as described by Zhang et al. (2020) in their study, highlights the interdependent relationship between the cerebral and cerebellar regions and the impact that cerebral lesions can have on cerebellar function. Baron originally proposed the concept of CCD in 1981 using a non-invasive continuous inhalation method of C^15^O_2_ and ^15^O_2_ combined with PET. This innovative approach allowed for the observation and measurement of reduced blood flow and metabolism in the contralateral cerebellum following a unilateral supratentorial cerebral lesion. Baron et al.’s (1981) study provided valuable insights into the phenomenon of CCD and its implications for understanding the functional connectivity between the cerebral and cerebellar regions.

The advancement of imaging technology has facilitated a growing body of research dedicated to the study of CCD. In their study, Reesink et al. (2018) utilized ^18^F-fluorodeoxyglucose positron emission tomography (FDG-PET) and ^11^C-Pittsburgh Compound B positron emission tomography imaging techniques to identify CCD in patients diagnosed with AD. Tripathi et al. (2022) employed the utilization of FDG-PET to examine CCD in a cohort of 18 patients diagnosed with Alzheimer’s disease. Their study revealed notable reductions in metabolic activity within the left temporal region and occipital region, as well as in the right cerebellum. These findings provide valuable insights into the metabolic alterations occurring in both the cerebral and cerebellar regions in patients with Alzheimer’s disease, further emphasizing the significance of CCD in neurodegenerative disorders (Tripathi et al., 2022). Zhang et al. (2023) employed MRI in conjunction with DTI to investigate patients with CCD following unilateral supratentorial subacute cerebral hemorrhage. Their findings revealed a significant correlation between hemodynamic alterations in the cortico-pontocerebellar-cerebellar fiber pathway and the development of CCD in these patients. These results offer valuable insights into the underlying pathological mechanisms of CCD, contributing to a more comprehensive understanding of this condition (Zhang et al., 2023). To ascertain the most effective diagnostic technique for CCD, Tripathi conducted a comprehensive evaluation of PET and MRI modalities. The study encompassed eight articles comprising a total of 1,246 subjects, with six studies utilizing PET imaging and two employing MRI and hybrid imaging. The findings revealed that PET demonstrated superior diagnostic efficacy in detecting CCD when compared to MRI. Furthermore, PET exhibited greater prognostic utility in predicting the occurrence of CCD (Tripathi et al., 2023).

As far back as, Tien and Ashdown (1992) reported the presence of cerebellar atrophy in a substantial number of patients diagnosed with CCD. These patients often exhibited notable contralateral supratentorial hemisphere atrophy as well (Tien and Ashdown, 1992). Gupta et al. (2015) documented two cases of patients diagnosed with Dyke-Davidoff-Masson syndrome accompanied by CCD. MRI findings revealed left cerebral hemisphere volume reduction and right cerebellar atrophy in both cases. Remarkably, despite the presence of cerebellar atrophy, neither patient exhibited any discernible signs of cerebellar dysfunction (Gupta et al., 2015). Kelly and Strick (2003) employed the retrograde trans-neuronal transport method using rabies virus and the prograde trans-neuronal transport approach with herpes simplex virus strain H129. Through these techniques, they elucidated that the primate cerebellum projects to the same regions of the cerebral cortex that it receives input from (such as primary motor cortex areas and dorsolateral prefrontal cortex). This finding supports the notion that the cerebral-cerebellar loop constitutes a closed loop circuit (Kelly and Strick, 2003). Despite the presence of a closed-loop circuit between the cerebrum and the cerebellum, the lack of apparent symptoms in the presence of cerebellar atrophy poses an intriguing inquiry warranting further investigation. The cerebellum is intricately interconnected with the brain through a diverse array of fiber connections.

Lu et al. (2011) employed resting-state fMRI to elucidate that the integrity of the cortical-pontine-cerebellar pathway determines the functional integrity between the cerebrum and cerebellum. Middleton utilized herpes simplex virus type 1 as a tracer to demonstrate the involvement of the retrograde cerebellar-thalamic-prefrontal cortex pathway as the anatomical basis for the cerebellum’s role in spatial working memory (Middleton and Strick, 1994). The studies conducted by Lu and Middleton primarily focused on the impact of fiber connections between the cerebrum and cerebellum on function. While they provided valuable insights into the potential relationship between disruption of cerebrum-cerebellum fiber connections and the emergence of CCD, the specific nature of these fiber connections, whether crossed, ipsilateral, or bilateral, was not further elucidated in their investigations. Reesink et al. (2018) discovered that concurrent CCD in patients with AD exhibits a heterolateral pattern, indicating a predominance of left-sided cortical hypometabolism along with right-sided cerebellar CCD. There appears to be a notable inclination toward the involvement of the right cerebellum in AD patients displaying CCD. Additionally, as previously discussed in relation to cerebellar laterality, there is a tendency for the right cerebellum to be implicated in the regulation of language function. Therefore, it is reasonable to speculate that when AD patients manifest language dysfunction, it may be attributed to dysfunction in the right cerebellum, aligning with our earlier discourse on the involvement of the cerebellum in language function as discussed in the language chapter. Our literature review has thus far not revealed comprehensive neural network mechanisms that elucidate CCD. This paucity of understanding may be attributed to the limitations of current diagnostic methods for CCD, which necessitate the use of expensive instruments. Furthermore, CCD is not a commonly occurring comorbidity, making it challenging to gather sufficient population data. The intricacies of CCD, coupled with the complexity of theoretical review, have resulted in a dearth of research on CCD at present.

## 5. Future and prospects

The cerebrum and cerebellum are interconnected and work together as cooperative assemblies, rather than being separate entities. Cerebro-cerebellar neural network loops serve as the anatomical basis for the involvement of the cerebellum in higher cognitive functions. Current research has demonstrated that cerebellar damage often results in cognitive impairments in domains such as visuospatial abilities, attention, EF, working memory, and language. These deficits are likely attributed to disruptions in the intact cerebro-cerebellar neural network pathways associated with cognition. However, the precise role of the cerebellum in the construction of cognitive neurobrain networks remains unclear. The current body of research on cerebellar involvement in cognitive functions has some limitations. Firstly, many studies have small sample sizes, which may be due to the rarity of isolated cerebellar lesions and the predominance of single-center studies. Additionally, the research methods employed are relatively homogeneous, primarily relying on imaging techniques, which can be costly. These limitations highlight the need for larger-scale studies involving diverse populations and the exploration of alternative research approaches to deepen our understanding of the cerebellum’s role in cognitive processes. Although imaging methods are more intuitive to study the anatomy of brain networks, the study of specific brain networks is not sufficiently detailed in the temporal dimension. Event-related potentials (ERPs) are electrophysiological responses recorded from the scalp using EEG in response to specific stimuli or cognitive processes. The advantages of using ERP as a research tool are numerous. Firstly, ERPs are non-invasive, making them safe and well-tolerated by participants. Additionally, they offer high temporal resolution, allowing for precise measurement of the timing of cognitive processes in the brain. ERPs are also relatively low-cost compared to other neuroimaging techniques, making them accessible for research in various settings. Moreover, ERPs have demonstrated good reliability and are sensitive to individual differences, enabling the investigation of inter-individual variations in cognitive processing. Lastly, ERP studies can be conducted in multicenter settings, facilitating larger-scale investigations and increasing the generalizability of findings. Overall, ERPs provide a valuable and versatile tool for studying cognitive neural processing in the brain. ERPs have the potential to serve as valuable biomarkers in the medical profession, as they can provide detailed information about brain function that may not be captured by traditional clinical tools (Luck et al., 2011). However, it is important to note that there is a limited number of studies investigating ERPs specifically in relation to cerebellar cognition. This highlights the need for further research in this area to fully understand the role of ERPs in assessing cerebellar function and their potential clinical applications. By conducting more studies using ERPs in the context of cerebellar cognition, researchers can gain a deeper understanding of the cerebellum’s involvement in cognitive processes and explore the potential diagnostic and therapeutic implications of ERP-based assessments in patients with cerebellar disorders.

## Author contributions

PZ, LD, YO, QL, LC, HQ, JW, JZ, and XY performed the literature searches and contributed to draft versions of the manuscript. PZ and LD wrote and revised the final version of the manuscript. XY made important suggestions and Guidance on the writing and revision of the manuscript. All authors contributed to the article and approved the submitted version.
